# Electrospun Semi-Alicyclic Polyimide Nanofibrous Membrane: High-Reflectance and High-Whiteness with Superior Thermal and Ultraviolet Radiation Stability for Potential Applications in High-Power UV-LEDs

**DOI:** 10.3390/nano11081977

**Published:** 2021-07-31

**Authors:** Xinxin Zhi, Huasen Wang, Xinying Wei, Yan Zhang, Yuancheng An, Haoran Qi, Jingang Liu

**Affiliations:** 1 Beijing Key Laboratory of Materials Utilization of Nonmetallic Minerals and Solid Wastes, National Laboratory of Mineral Materials, School of Materials Science and Technology, China University of Geosciences, Beijing 100083, China; 3003200015@cugb.edu.cn (X.Z.); 2003200022@cugb.edu.cn (X.W.); 3003200016@cugb.edu.cn (Y.Z.); 2103190039@cugb.edu.cn (Y.A.); 2103200030@cugb.edu.cn (H.Q.); 2 POME Technology Co. Ltd., Liaocheng 252399, China; whs@pome.com.cn

**Keywords:** polyimide, electrospinning, nanofibrous membrane, reflectivity, UV-LED

## Abstract

Polymeric nanofibrous membranes (NFMs) with both high whiteness and high thermal and ultraviolet (UV) stability are highly desired as reflectors for ultraviolet light-emitting diodes (UV-LEDs) devices. In the current work, a semi-alicyclic and fluoro-containing polyimide (PI) NFM with potential application in such kinds of circumstances was successfully fabricated from the organo-soluble PI resin solution via a one-step electrospinning procedure. In order to achieve the target, a semi-alicyclic PI resin was first designed and synthesized from an alicyclic dianhydride, 3,4-dicarboxy-1,2,3,4,5,6,7,8-decahydro-1-naphthalenesuccinic dianhydride (or hydrogenated tetralin dianhydride, HTDA), and a fluoro-containing diamine, 2,2-bis[4-(4-amino-phenoxy)phenyl]hexafluoropropane (BDAF), via an imidization procedure. The derived PI (HTDA-BDAF) resin possessed a number-average molecular weight (*M*_n_) higher than 33,000 g/mol and was highly soluble in polar aprotic solvents, such as *N,N*-dimethylacetamide (DMAc). The electrospinning solution was prepared by dissolving the PI resin in DMAc at a solid content of 25–35 wt%. For comparison, the conventional high-whiteness polystyrene (PS) NFM was prepared according to a similar electrospinning procedure. The thermal and UV stability of the derived PI and PS NFMs were investigated by exposure under the UV-LED (wavelength: 365 nm) irradiation. Various thermal evaluation results indicated that the developed PI (HTDA-BDAF) NFM could maintain both the high reflectance and high whiteness at elevated temperatures. For example, after thermal treatment at 200 °C for 1 h in air, the PI (HTDA-BDAF) NFM exhibited a reflectance at a wavelength of 457 nm (*R*_457_) of 89.0%, which was comparable to that of the pristine PI NMF (*R*_457_ = 90.2%). The PI (HTDA-BDAF) NFM exhibited a whiteness index (*WI*) of 90.88, which was also close to that of the pristine sample (*WI* = 91.22). However, for the PS NFM counterpart, the *R*_457_ value decreased from the pristine 88.4% to 18.1% after thermal treatment at 150 °C for 1 h, and the sample became transparent. The PI NFM maintained good optical and mechanical properties during the high dose (2670 J/cm^2^) of UV exposure, while the properties of the PS NFM apparently deteriorated under the same UV aging.

## 1. Introduction

In the past decades, high-power light-emitting diodes (LEDs), such as ultraviolet LEDs (UV-LEDs), have been paid increasing attention in outdoor displays, lighting, automobile indicators, water disinfection, and other high-tech applications [1,2,3]. In order to achieve a lightweight and low-cost design for UV-LEDs, polymeric materials have been highly suggested to be used in the fabrication of devices as reflector cups, encapsulant, and so on [4]. For example, polymeric nanofibrous membranes (NFMs) with high whiteness and high reflectivity have recently been investigated as candidates for reflector cups used in high-performance LED devices. Tang and coworkers reported high-whiteness poly(lactic-co-glycolic acid) nano-fibrous membranes based on the electrospinning procedure [5]. The developed membranes showed high reflective and scattering abilities and efficiently improved the luminous efficiency of the fabricated white LEDs. Recently, the construction of polymeric NFMs was greatly promoted by the biomimetic methodology from natural organisms. In 2009, Yip et al. fabricated brilliant-whiteness surfaces from electrospun nanofiber webs by the inspiration from Cyphochilus white beetles living in Southeast Asia [6]. Similarly, Syurik et al. fabricated large-scale and highly scattering surfaces with exceptional whiteness from poly(methyl methacrylate) films by the Cyphochilus-inspired idea [7]. Inspired by natural organisms with high whiteness, Zeighami et al. developed optically efficient nanofiber coatings with extra high whiteness and opaqueness via this biomimetic procedure [8]. Polyamide (nylon) and polyacrylonitrile nanofiber layers were coated on the surface of a paper using an electrospinning method. High whiteness and complete opaqueness were achieved even on black cardboard by means of a thin layer of the electrospun nanofibers. Very recently, Toivonen et al. fabricated high-whiteness membranes by controlling the light transport in the membranes of cellulose nanofabrics via tuning the porosity and morphological features of the polymers [9].

Although various polymeric ultrafine NFMs have been investigated as components for LED applications, the lifetime issue of polymers has to be addressed in the case of high-energy UV-LEDs. For example, after a period of time of operation in UV-LEDs, the UV light and heat energy generated by LED materials might deteriorate the properties of the common polymers [10]. On the one hand, overheating might cause the creep or melt of polymeric materials, resulting in the change in thickness or volume of the components. The induced nonuniformities in the polymer components will undoubtedly deteriorate the reliability and lifetime of the UV-LEDs. On the other hand, the high-energy short-wavelength UV irradiation might deepen the color of the polymer components, which might deteriorate the emission efficiency of the UV-LEDs. As UV-LEDs are usually operated at significantly elevated temperatures, the thermal and UV degradation of polymeric components are often accelerated and enhanced during the applications. The change in color and intensity of the polymer components is disadvantageous for the long-term operation of UV-LEDs. Although the influence of the UV-LED irradiation on the property deterioration of polymer components could be alleviated by increasing the distance of the polymers from the LED sources by structural design, polymeric NFMs with high UV resistance, low yellowness, high whiteness (high reflectivity), and thermal stability are highly desired for the R&D of high-performance UV-LEDs [11]. Unfortunately, the availability of polymeric materials possessing both high thermal and UV stability and high whiteness (high reflectivity) is limited because the methodologies improving the thermal and UV stability and the whiteness of one specific polymer are usually contradictory. For example, highly conjugated aromatic components usually afford polymers good thermal and UV stability; however, the existence of the inter- or intramolecular charge transfer (CT) interactions caused by the conjugated molecular structure usually deteriorate the whiteness and optical reflectivity of the polymers [12].

Polyimides (PIs) have been widely used in a variety of applications in which high thermal and environmental stability is required [13]. The excellent combined thermal, mechanical, electrical, and dielectric properties make PIs good candidates for various high-tech applications [14,15,16,17,18,19,20]. However, the intrinsic deep colors and low whiteness of the standard wholly aromatic PI films or fibrous membranes caused by the CT interactions in the molecular structures greatly limit their wide applications in optoelectronic areas [21,22,23,24,25]. In view of the property limitation of conventional wholly aromatic PIs in optoelectronics applications, various modifications have been performed in past decades, including the incorporation of nonconjugated alicyclic or aliphatic moieties, highly electronegative substituents, and asymmetrical or bulky units. In our previous work, a series of PI NFMs with high whiteness (whiteness index > 90) and high reflectivity (>90 at the wavelength of 427 nm) were developed from semi-alicyclic PIs for potential applications for standard LEDs [26,27]. However, the behaviors of the derived semi-alicyclic PI ultrafine NWFMs in UV-LED radiation environments were not investigated. To the best of our knowledge, there have been few reports on the subject in the literature up to now.

In the current work, as part of our continuous effort to develop high-performance PI NFMs for advanced optoelectronics applications, a novel PI NFM was fabricated via a one-step electrospinning procedure from the newly developed organo-soluble PI resin derived from an alicyclic dianhydride and a fluoro-containing aromatic diamine. The designed PI (HTDA-BDAF) possessed many desirable characteristics as the starting material for the target applications. First, the synergistic effects of the nonconjugated alicyclic units in the dianhydride moiety and the bulky hexafluoroisopropylidene units in the diamine moiety will enhance the soluble-processability of the PI resin, which is beneficial for the quality of the electrospun PI NFMs [26]. Secondly, these synergistic effects are helpful for reducing the charge transfer (CT) interactions from the electron-donating diamine units to the electron-withdrawing dianhydride units; thus, it might prohibit the coloration of the PI NFMs [27]. The influence of the molecular structure of the PI ultrafine NFM on their thermal stability, optical parameters, and UV radiation resistance was studied in detail. For comparison, a standard high-whiteness and highly reflective polystyrene (PS) ultrafine NFM and a standard wholly aromatic PI membrane [poly(pyromellitic anhydride-co-4,4′-oxydianiline)] (PI-ref) were fabricated at the same time. The comprehensive properties of the newly designed PI ultrafine NFM were compared with the PS and PI-ref counterparts.

## 2. Materials and Methods

### 2.1. Materials

3,4-Dicarboxy-1,2,3,4,5,6,7,8-decahydro-1-naphthalenesuccinic dianhydride or hydrogenated tetralin dianhydride (HTDA) was kindly supplied by Newera Kesense New Materials Co. Ltd., Shandong, China and dried at 150 °C in vacuo for 24 h prior to use. Pyromellitic anhydride (PMDA) was obtained from HOPE Co. Ltd. (Shijiazhuang, China) and dried at 180 °C in vacuo for 24 h prior to use. 4,4′-Oxydianiline (ODA) and 2,2′-bis[(4-aminophenoxy)phenyl]hexafluoropropane (BDAF) were purchased from Tokyo Chemical Industry (TCI) (Tokyo, Japan) and used as received. Polystyrene (PS, average molecular weight: 35,000 g mol^−1^) was purchased from Sigma-Aldrich (Shanghai, China) and used as received. *N,N*-Dimethylacetamide (DMAc), *N,N*-dimethylformamide (DMF), γ-butyrolactone (GBL), and other solvents were purchased from Beijing Yili Fine Chemicals (Beijing, China) and purified by distillation prior to use. The other commercially available reagents were used without further purification.

### 2.2. Measurements

Inherent and absolute viscosities of the PI (HTDA-BDAF) resin were tested with an Ubbelohde viscometer (As-one, Osaka, Japan) with a 0.5 g dL^−1^ NMP solution at 25 °C and a Brookfield DV3TRVCP viscometer (Middleboro, MA, USA) at 25 °C, respectively. Molecular weights of the PI resin, including number average molecular weight (*M*n) and weight average molecular weight (*M*w), were tested via a gel permeation chromatography (GPC) system (Shimadzu, Kyoto, Japan). The solubility of the PI resin in organic solvent was detected by immersing 1.0 g of the PI resin into 9.0 g of the tested solvent with a solid content of 10 wt%. The mixture was stirred at room temperature for 24 h. Then, the solubility behaviors of the PI resin in the solvent were classified visually as three grades: completely soluble (++), partially soluble (+−), and insoluble (−).

An Iraffinity-1S FT-IR spectrometer (Shimadzu, Kyoto, Japan) was used to test the attenuated total reflectance–Fourier-transform infrared (ATR–FTIR) spectra of the PI NFM. A Hitachi U-3210 spectrophotometer (Tokyo, Japan) was used to test the ultraviolet-visible (UV-Vis) reflectance spectra of the PI NFMs. A JSM-6700F (JEOL, Tokyo, Japan) field-emission scanning electron microscopy (FE-SEM) system with an accelerating voltage of 15 kV was used to observe the micro-morphologies of the PI NFMs. An X-rite color i7 spectrophotometer (Grand Rapids, Michigan, USA) was used to determine the CIE Lab optical parameters of the PI NFMs according to ASTM D1925 “Test method for yellowness index of plastics.” L* is the lightness, where 100 is white and 0 is black; a positive a* indicates a red color and a negative one indicates a green color; a positive b* indicates a yellow color and a negative one indicates a blue color. The whiteness values (*WI*) of the PI membranes were calculated with Equation (1) [26].

*WI* = 100 − [(100 − L*)^2^ + a*^2^ + b*^2^]^1/2^ (1)

where *WI* stands for whiteness, L* stands for index of lightness, and a* and b* stand for chromaticity coefficient.

A TA-Q50 thermal analysis system (New Castle, Delaware, USA) was used to perform the thermogravimetric analysis (TGA) at a heating rate of 20 °C min^−^^1^ in nitrogen. A TA-Q 100 thermal analysis system (New Castle, Delaware, USA) was used to test the differential scanning calorimetry (DSC) of the PI NFMs at a heating rate of 10 °C min^−^^1^ in nitrogen.

**Figure 1:**
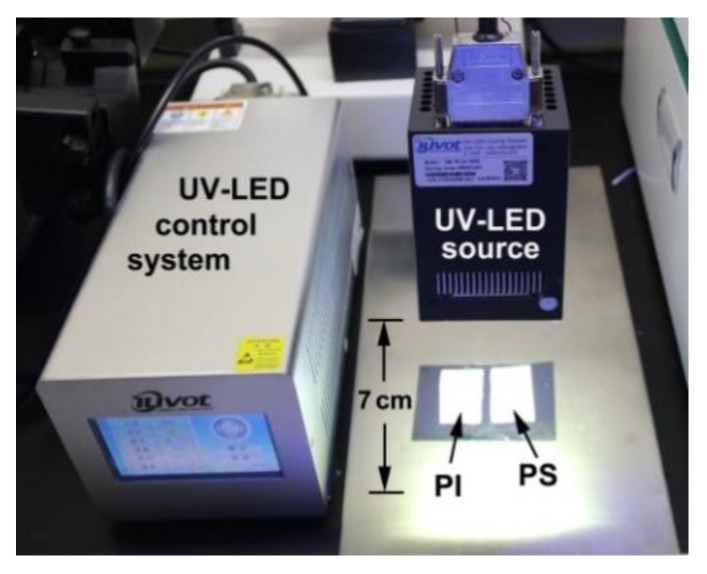
UV-LED exposure apparatus and measurement conditions.

The UV-LED aging test was performed with a UV-LED (wavelength: 365 nm) system (Model: IMS-811A-0606, IUVOT Co. Ltd. Jiangsu, China) with the conditions shown in Figure 1. The distance between the UV-LED array source and the polymers, including PI and the referenced polystyrene (PS) NFM samples, was controlled to be 7.0 ± 0.5 cm in order to avoid the heat impact of the UV source on the thermally unstable PS sample. The UV exposure time was set to be 30 min (total exposure doze: ~2670 J/cm^2^).

### 2.3. Synthesis of PI Resin

Into a 250 mL three-necked flask equipped with a mechanical stirrer and a nitrogen inlet, the diamine BDAF (5.1845 g, 0.01 mol) was first dissolved in newly distilled DMAc (20 g). Then, the dianhydride HTDA (3.0631 g, 0.01 mol) and additional DMAc (4.7 g) were added into the homogeneous diamine solution, producing a reaction system with a solid content of 25 wt%. After stirring at room temperature for 24 h, a mixture of acetic anhydride (5.1 g, 0.05 mol) and pyridine (3.2 g, 0.04 mol) was then added. The reaction solution was stirred at room temperature for another 24 h. Then, the obtained viscous solution was carefully poured into an excess of ethanol to yield white resin. The resin was filtered and dried at 80 °C in vacuum for 24 h (Yield: ~96%). The well-dried PI resin was redissolved into DMAc with a solid content of 10 wt%. Then, the PI solution was filtered through a 0.45 μm Teflon filter to remove any impurities. Next, the PI solution was slowly poured into the highly pure ethanol to afford the PI resin.

FTIR (cm^−1^): 2931, 1782, 1712, 1601, 1502, 1383, 1242, 1203, 1171, 1016, 968, 930, and 829. ^1^H NMR (DMSO-*d*_6_, ppm): 7.39–7.38 (*m*, 4H), 7.30–7.29 (*m*, 4H), 7.23–7.21 (*m*, 4H), 7.15–7.13 (*m*, 4H), 3.18–3.16 (*m*, 1H), 3.08–3.01 (*m*, 1H), 2.91–2.85 (*m*, 1H), 2.32–2.15 (*m*, 3H), 2.02–1.92 (*m*, 2H), 1.78–1.67 (*m*, 2H), and 1.58–1.05 (*m*, 8H).

### 2.4. Fabrication of PI NFM via Electrospinning

Four PI NFMs (PI-1~PI-4) were fabricated via the electrospinning procedure with the tailored solid contents of the PI solutions, the electrospinning voltage, and the inner diameters (IDs) of the spinnerets, as shown in Table 1. Four PI membranes with different micro-morphologies and fiber diameters were prepared by adjusting the solution and electrospinning parameters. The four PI NFMs were denoted as PI-1, PI-2, PI-3, and PI-4, respectively, as shown in Table 1. The fully dried PI (HTDA-BDAF) resin was dissolved in DMAc at room temperature with different solid contents of 25–35 wt% to obtain the electrospinning solution. The PI solution was loaded into a 5 mL syringe and squeezed out through a spinneret by a syringe pump at a speed of 0.2 mL/h. The ID values of the spinneret were 0.21 or 0.50 mm. Different voltages of 12–18 kV were applied between the syringe and the rolling drum collector. The distance between the spinneret and the collector (alumina foil) was 15 cm. Random aligned PI fibers were deposited on the aluminum foil located on the rotating drum collector (speed: 200 rpm). The temperature and relative humidity during electrospinning were fixed at about 20 ± 2 °C and 30 ± 5%, respectively.

The electrospinning parameters of the polystyrene (PS) NFM were the same as those mentioned above. For comparison, a poly(pyromellitic dianhydride-co-4,4′-oxydianiline) (PI-ref, PMDA-ODA) NFM was prepared via a two-step electrospinning procedure with soluble poly(amic acid) (PAA) as a starting material, followed by the high-temperature imidization procedure up to 350 °C [28].

## 3. Results and Discussion

### 3.1. PI Resin Synthesis and Electrospun PI NFM Preparation

**Figure 2:**
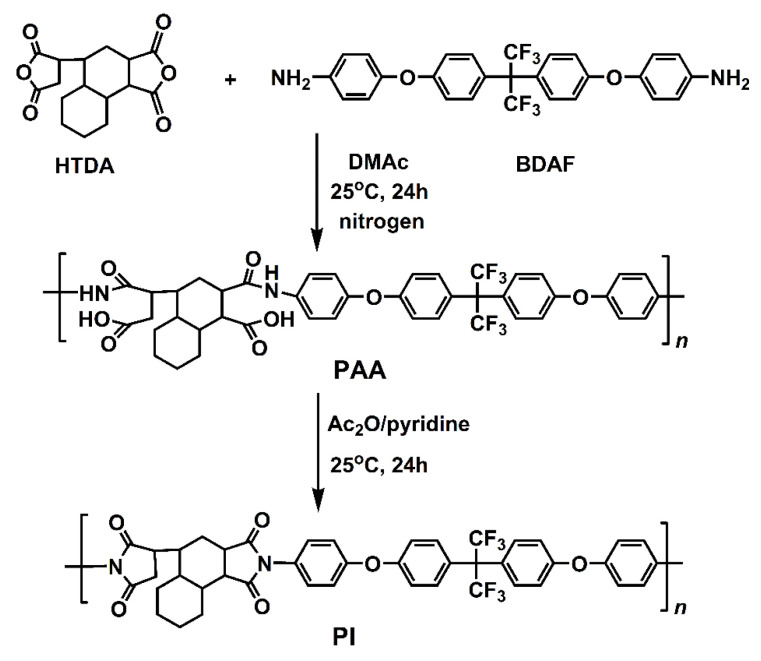
Synthesis of PI (HTDA-BDAF) resin.

A newly designed PI (HTDA-BDAF) was synthesized via a two-step imidization procedure, as illustrated in Figure 2. An alicyclic dianhydride HTDA and a fluoro-containing diamine, BDAF, were used as the monomers for the PI synthesis. The nonconjugated alicyclic units in HTDA and the large electronegativity of the C-F bonds in BDAF are anticipated to be able to efficiently prohibit the charge transfer (CT) interactions between the electron-donating diamine moiety and the electron-accepting dianhydride moiety [21]. This CT interaction was thought to be one of the main reasons inducing the coloration of conventional wholly aromatic PIs [29]. As expected, the polymerization reaction for the PI was stable and a homogeneous PI solution was obtained after the reaction. Fine and white PI resins were almost quantitatively obtained.

The PI resin showed an inherent viscosity ([*η*]_inh_) of 0.63 dL g^−^^1^, a number average molecular weight (*M*_n_) of 3.38 × 10^4^ g mol^−^^1^, and a weight average molecular weight (*M*_w_) of 5.76 × 10^4^ g mol^−^^1^, respectively, as shown in Table 2. It can be deduced from the moderate inherent viscosity and molecular weights of the PI resin that the alicyclic HTDA dianhydride showed good reactivity during the polymerization with BDAF. In addition, the PI resin exhibited a narrow PDI value of 1.70, indicating that rare side reactions occurred in the polymerization. The solubility of the PI resin in various solvents was detected and the results are shown in Table 2. It was easily soluble in the polar aprotic solvents, including NMP, DMAc, and DMF at room temperature, and partially soluble in less polar chloroform and THF. The good solubility of the current PI resin was mainly attributed to the less conjugated molecular structure and low molecular packing density caused by the perhydro-naphthalene and bulky hexafluoroisopropylene units in the polymer. The excellent solubility of the PI resin makes it possible to use PI solutions to fabricate electrospun PI NFMs, which is helpful in reducing the coloration of the PI NFMs.

**Figure 3:**
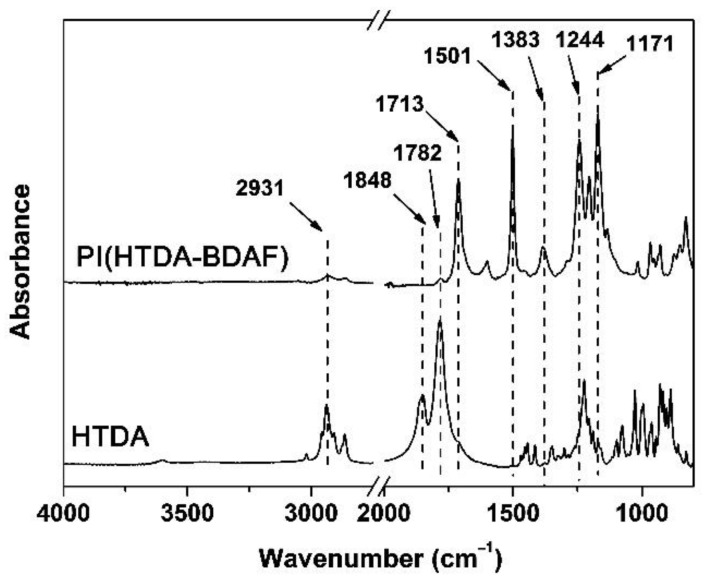
ATR-FTIR spectra of the PI (HTDA-BDAF) and the HTDA dianhydride.

Figure 3 shows the ATR-FTIR spectra of the PI and the starting HTDA dianhydride. It can be clearly observed that the characteristic absorptions of anhydride carbonyl in HTDA, especially the asymmetrical one at 1848 cm^−1^, totally disappeared in the spectrum of PI. The symmetrical absorption of anhydride carbonyl in HTDA at 1782 cm^−1^ was overlapped with the characteristic absorptions of the imide rings. For the PI resin, the asymmetrical and symmetrical carbonyl stretching vibrations at the wavenumbers of 1782 cm^−1^ and 1713 cm^−1^, respectively, and the C-N stretching vibration at 1383 cm^−1^ were clearly observed. Meanwhile, the characteristic absorptions of saturated C-H bonds in the HTDA moiety at 2931 cm^−1^ were detected in both spectra. However, the characteristic absorptions of unsaturated C=C bonds at 1501 cm^−1^ and those of the C-F bonds at 1171 cm^−1^ were only detected in the spectrum of PI. The spectral information was in good consistency with the structural features of the PIs.

**Figure 4:**
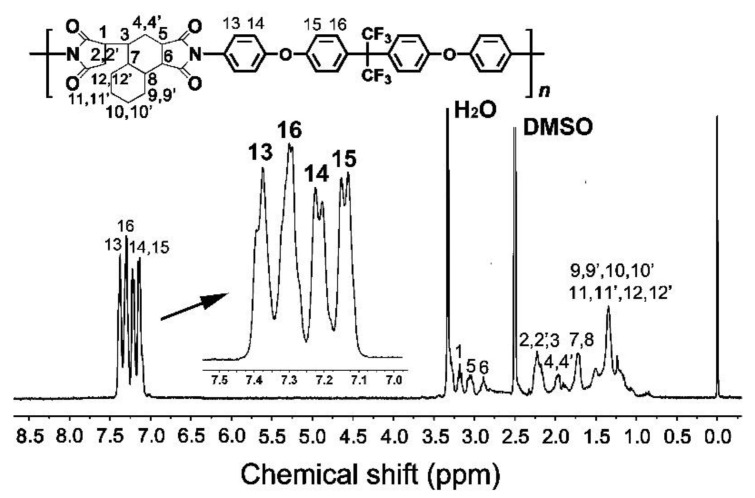
^1^H NMR spectrum of the PI(HTDA-BDAF) resin (NMR solvent: DMSO-*d*_6_).

Figure 4 depicts the ^1^H-NMR spectrum of the PI resin. The absorptions of the protons in the PI were clearly divided into two parts. The absorptions of the aromatic protons (H_13_~H_16_) in the BDAF appeared at the downfield area in the spectrum (7.0–7.5 ppm), whereas the aliphatic protons (H_1_~H_12_) in the HTDA moiety revealed the absorptions at the upfield region in the spectrum (1.0–3.5 ppm). This is also in good consistency with the theoretical molecular structure of the PI.

**Figure 5:**
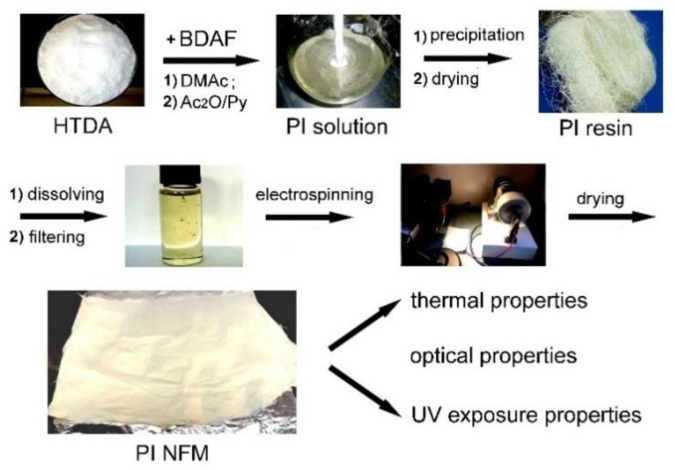
Preparation of PI NFM via electrospinning procedure from soluble PI resin.

**Figure 6:**
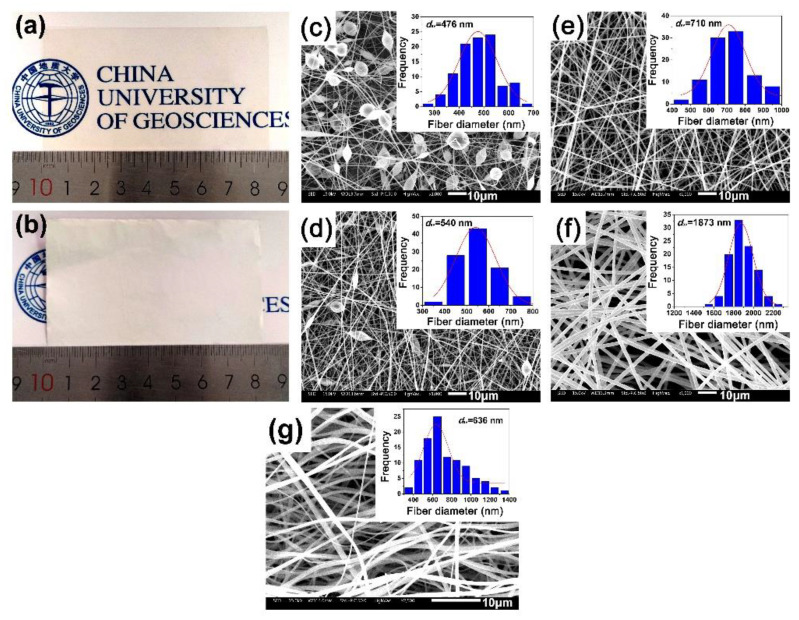
Macroscopic and microscopic morphology of PI samples. (**a**) Appearance of PI film; (**b**) appearance of PI membranes; (**c**–**f**) SEM images of PI-1, PI-2, PI-3, and PI-4 membranes, respectively; (**g**) SEM image of PI-ref NFM.

A series of PI (HTDA-BDAF) NFMs were fabricated by the electrospinning procedure, as shown in Figure 5, with the tailored solid contents of the starting PI solution and the electrospinning parameters shown in Table 1. In order to understand the effects of the molecular structure on the properties of the PIs, the PI (HTDA-BDAF) film was also prepared with a procedure according to our previous work [30]. The appearances of the free-standing PI film and NFM are shown in Figure 6a,b, respectively. It could be clearly observed that the PI film exhibited a very pale-yellow appearance with excellent optical transparency, and the opaque PI NFMs showed high whiteness. The microscopic morphologies of the PI NFMs were investigated by SEM measurements and the results are shown in Figure 6c–f. For comparison, the SEM image of the PI-ref NFM is shown in Figure 6g. The results indicated that PI nonwoven NFMs with random aligned fibers were all successfully prepared, although they showed different fiber morphologies. As expected, for the PI-1 NFM derived from PI solution with low a solid content (25 wt%) and low electrospinning voltage (12 kV), although the electrospun NFM with fine fibers could be obtained, bead-like structures were observed on the fibers, as shown in Figure 6c. This is mainly due to the relatively low viscosity of the starting PI solution. The formation of bead structures was gradually reduced by increasing the solid contents of the PI solution and adjusting the electrospun parameters, as shown in Figure 6d for PI-2, 6e for PI-3, and 6f for PI-4. The average fiber diameters (*d*_av_) of the PI NFMs increased with the solid contents of the PI solutions. Thus, the optimal electrospinning parameters for the current PI NFMs included a solid content of 30 wt%, an electrospinning voltage of 15 kV, and a spinneret inner diameter of 0.21 mm. Under these conditions, PI-3 NFM with the smooth and fine fibers (*d*_av_ = 710 nm) was obtained.

### 3.2. Optical Properties

**Figure 7:**
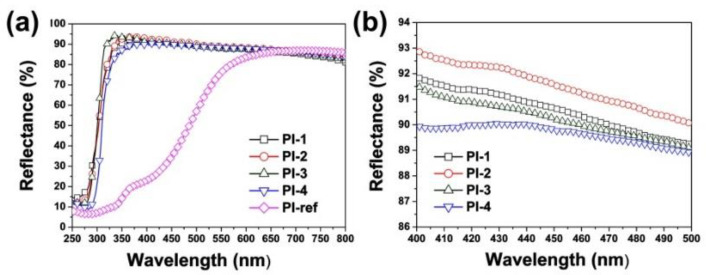
Optical properties of electrospun PI membranes. (**a**) UV-Vis reflectance plots of PI membranes in the range of 250~800 nm; (**b**) UV-Vis reflectance plots of PI membranes in the range of 400~500 nm.

In order to reveal the influence of the chemical structure and physical morphologies of the PI NFMs on the optical properties, the ultraviolet-visible light (UV-Vis) reflectance spectra and CIE Lab optical parameters of the PI NFMs were investigated. For the current applications, a higher UV-Vis reflectance and higher whiteness index (*WI*) for the PI NFMs are preferred in order to achieve a high efficiency. Figure 7 shows the UV-Vis reflectance spectra of the PI and PI-ref NFMs, and the reflectance values of the PI NFMs at the wavelength of 457 nm (*R*_457_) are listed in Table 3. It can be clearly observed from Figure 7a that the currently developed PI (HTDA-BDAF) NFMs showed much higher *R*_457_ values than that of the PI-ref NFM. For example, PI-3 NFM had a *R*_457_ value of 90.2% at room temperature, which was nearly 53.0% higher than that of the PI-ref NFM. It can be mainly attributed to the prohibited formation of the charge transfer complexes (CTC) in the molecular structures of the PI due to the existence of the nonconjugated alicyclic units in the HTDA moiety and the highly electronegative hexafluoroisopropylidene units in the BDAF moiety. Effects of the micro-morphologies of the PI NFMs on the optical reflectance could also be deduced from Figure 7b. The *R*_457_ values of the PI NFMs decreased with the order of PI-2 (91.3) > PI-1 (90.4) > PI-3 (90.2) > PI-4 (89.5). The thinner fiber diameters and the bead structures in PI-1 and PI-2 were beneficial for increasing the *R*_457_ values of the PI NFMs. For PI NFMs consisting of fine and smooth fibers, the smaller the average diameter of the fiber in the PI NFM, the higher the reflectance of the NFM to the light. PI-3 NFM exhibited a higher *R*_457_ value than that of PI-4, which is associated with the higher specific surface area of the former with a smaller *d*_av_ value.

**Figure 8:**
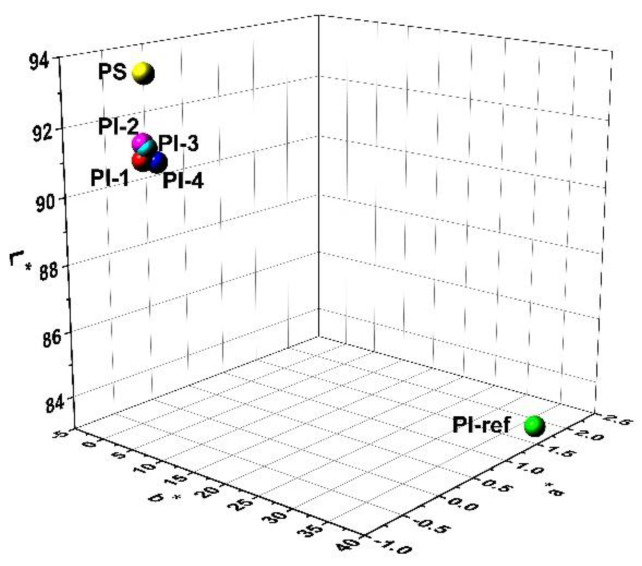
Three-dimensional (3D) plots of the CIE Lab parameters for electrospun PI, PI-ref, and PS membranes.

The CIE Lab optical parameters of the PI and PI-ref NFMs were compared and the results are shown in Figure 8. From the three-dimensional (3D) plots, one can clearly observe that the newly developed PI NFMs showed a much higher lightness (L*) and much lower yellow indices (b*) than those of the PI-ref NFM. However, the currently developed PI NFMs showed inferior optical parameters than those of the PS NFM, which was usually thought to be the standard high-whiteness polymer fabric. The polymer NFMs showed decreased whiteness indices (*WI*) with the order of PS (93.26) > PI-3 (91.22) > PI-ref (59.00) according to the data shown in Table 3. This trend is in good consistency with the structural features of the polymers. For PS, the bulky and pendant phenyl side chains efficiently decreased the crystallinity of the molecular chains and improved the optical reflectance of the NFM. For PI (HTDA-BDAF), the synergistic effects of nonconjugated perhydro-naphthalene and highly electronegative -C(CF_3_)_2_ groups endowed the NFM with good optical reflectance. Contrarily, the wholly aromatic and highly conjugated molecular structures of the PI-ref greatly deteriorated the optical properties of the derived NFM.

Besides the chemical factors that affect the whiteness of the polymer NFMs, the whiteness of polymer NFMs is also affected by the physical structure of the mats. It has been well established that the whiteness of the surfaces of Cyphochilus beetles and the poplar leaves is mainly caused by the disordered fiber structure [6,7]. It could be deduced from the appearance of the PIs shown in Figure 6 that the PI film was transparent, while the PI NFM was opaque and showed brilliant whiteness. This indicated that fiber structure is an important factor affording the high-whiteness surfaces.

### 3.3. Thermal Properties

**Figure 9:**
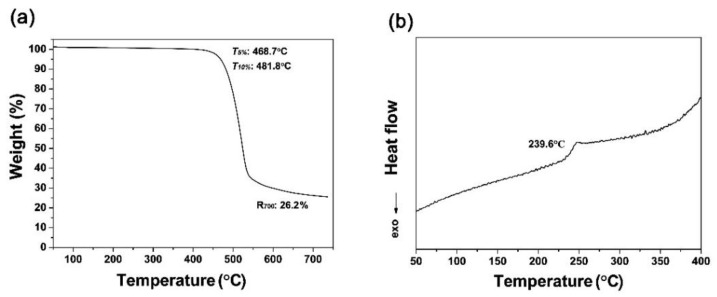
Thermal properties of PI-3 membrane. (**a**) TGA; (**b**) DSC.

Although the PI (HTDA-BDAF) NFMs showed inferior optical properties than those of the PS NFM analog, they exhibited a much better thermal resistance than that of the PS NFM, as could be deduced from various thermal property evaluations. For the current applications, higher thermal stabilities for the polymers are highly preferred in order to achieve a high reliability and safety. The poor thermal stability of the commercially available high-whiteness polymer materials, such as PS, polylactic acid (PLA), and polyamide (nylon), is one of the key factors limiting their wide applications in high-temperature optoelectronic fields, such as the reflectors for high-power LED devices. The research and development of polymer materials with both high whiteness and high thermal stability is highly required for the high-tech applications. As PI stands for a class of thermally stable polymer, we expected that the PI NFMs might have excellent thermal stability. The thermal stability of PI NFMs was evaluated by thermogravimetric analysis (TGA) and differential scanning calorimetry (DSC) measurements, and the results are shown in Figure 9. The representative PI-3 NFM showed a glass transition temperature (*T*_g_) as high as 239.6 °C, a 5% weight loss temperature (*T*_5%_) of 468.7 °C, and a residual weight ratio at 700 °C (*R*_700_) of 26.2% in nitrogen.

**Figure 10:**
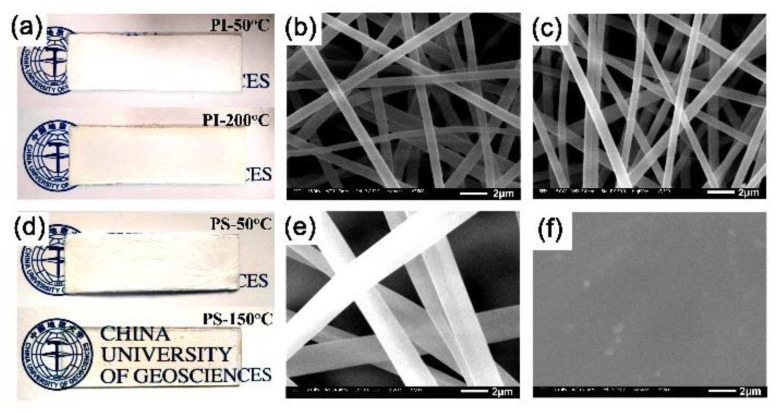
Thermal stability of the high-whiteness membranes. (**a**) Appearance of PI-3 membrane after heat treatment (upper: PI-50 °C; lower: PI-200 °C); (**b**) SEM image of PI-3 membrane after thermal treatment at 50 °C for 1 h; (**c**) SEM image of PI-3 membrane after thermal treatment at 200 °C for 1 h; (**d**) Appearance of PS membranes after heat treatment (upper: PS-50 °C; lower: PS-150 °C); (**e**) SEM image of PS membrane after thermal treatment at 50 °C for 1 h; (**f**) SEM image of PS membrane after thermal treatment at 150 °C for 1 h.

**Figure 11:**
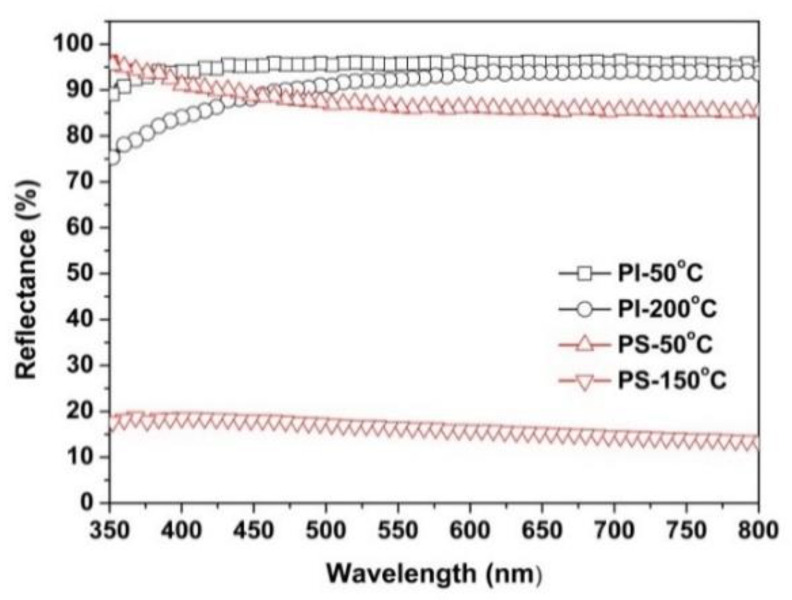
UV-Vis reflectance plots of PI-3 membranes after heat treatment.

To further study the retention ability of whiteness level of the PI NFM at elevated temperatures, thermal treatments for PI-3 NFM and the referenced PS NMF were performed. The PI and PS NFMs thermally treated at different temperatures were named as PI-50 °C, PI-200 °C, PS-50 °C, and PS-150 °C, respectively, as shown in Table 3. The changes in appearances and microscopic morphologies of the polymer NFMs after heating treatment are shown in Figure 10. The UV-Vis reflectance spectra of the polymer NFMs after thermal treatment are shown in Figure 11. The corresponding optical data of the polymer NFMs after thermal treatment are tabulated in Table 3. It could be clearly observed from Figure 10a,d that the PI-3 NFM basically maintained the original whiteness even after thermal treatment at 200 °C for 1 h. The PI-3 NFM showed *R*_457_ values of 90.2% for the pristine sample, 95.7% for PI-50 °C, and 89.0% for PI-200 °C. Meanwhile, the PI-3 NFM showed *WI* values of 91.22 for the pristine sample, 93.46 for PI-50 °C, and 90.88 for PI-200 °C. Thus, the thermal treatment at a relatively lower temperature (50 °C) could increase the optical properties of the PI-3 NFM, which might be due to the releasing of the absorbed moisture in PI-3 NFM during the thermal treatment at 50 °C. Thermal treatment at an elevated temperature (200 °C) slightly deteriorated the optical properties of the PI NFM. For the PS NFM sample, although the thermal treatment process at 50 °C did not apparently affect the optical properties and micro-morphologies of the PS NFM (Figure 10e), the higher-temperature treatment at 150 °C nearly totally destroyed the fibrous structure of the polymer, as evidenced by Figure 10d,f. The PS NFM showed *R*_457_ values of 88.4% for the pristine sample, 88.1% for PS-50 °C, and 18.1% for PS-150 °C, as can be seen from Figure 11. Thus, the PS NFM exhibited a much poorer high-temperature resistance compared with that of the currently developed PI NFM. This phenomenon happened because of a relatively low *T*_g_ of PS (~100 °C) [31].

### 3.4. UV Exposure Properties

**Figure 12:**
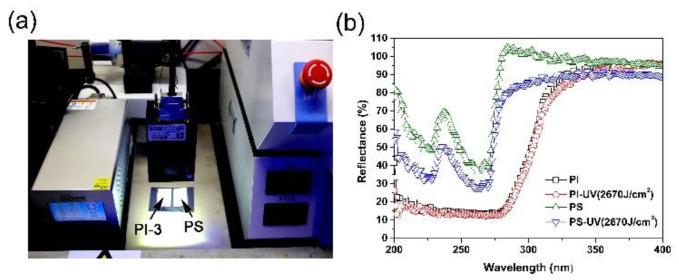
UV-Vis plots of polymer membranes after UV radiation (365 nm) treatment. (**a**) UV exposure test; (**b**) UV-Vis reflectance plots in the wavelength range of 200~400 nm.

The high thermal stability and high-whiteness features of the currently developed semi-alicyclic PI NFMs make them good potential candidates as reflectors for high-power LED devices. For such kind of applications, another important property is the UV exposure stability. A significant degradation in molecular structures or optical properties might occur after UV irradiation for many polymers. Therefore, UV stability tests for the polymer NFMs were carried out, and the result is shown in Figure 12. When the PS NFM was subjected to the UV irradiation in air, it underwent a rapid yellowing and a gradual embrittlement. The reflectance of the PS NFM apparently decreased after UV exposure. For the PI NFM, the reflectance value of the PI NFM nearly maintained the same level after UV exposure with a high dose of 2670 J/cm^2^, indicating the higher UV stability of the PI NFMs. Meanwhile, the PI NFM maintained the flexible and tough nature.

## 4. Conclusions

Semi-alicyclic and fluoro-containing PI NFM was designed and prepared in order to meet the requirements of high-power UV-LEDs. The synergistic effects of the alicyclic and fluoro-containing groups endowed the derived PI-3 (HTDA-BDAF) NFM with high whiteness (*WI* = 91.22 at room temperature; *WI* = 90.88 after thermal treatment at 200 °C for 1 h in air), high UV reflectance (*R*_457_ = 90.2% at room temperature; *R*_457_ = 89.0% after thermal treatment at 200 °C for 1 h in air), good thermal stability (*T*_g_ = 239.6 °C), and good UV exposure stability. Comparatively, the standard high-whiteness PS NFM analogs lost the fiber structure only after thermal treatment at 150 °C for 1 h. The good comprehensive properties of the currently developed PI NFMs are quite beneficial for their applications as reflectors or other optical components for high-power UV-LED devices.

## Figures and Tables

**Table 1:** Electrospinning parameters of PI NFMs.

	Solid Content (wt%)	Voltage Supply (kV)	Spinneret ID (mm)
PI-1	25	12	0.50
PI-2	30	15	0.50
PI-3	30	15	0.21
PI-4	35	18	0.50

**Table 2:** Inherent viscosities, molecular weights, and solubility of PI resins.

Sample	[*η*]_inh_ ^1^ (dL/g)	Molecular Weight ^2^ (×10^4^ g/mol)			Solubility ^3^				
		*M*_n_	*M*_w_	PDI	NMP	DMAc	DMF	CHCl_3_	THF
PI (HTDA-BDAF)	0.63	3.38	5.76	1.70	++	++	++	+−	+−

^1^ Inherent viscosity measured with PI resins at a concentration of 0.5 g dL^−^^1^ in NMP at 25 °C; ^2^ *M*_n_: number average molecular weight; *M*_w_: weight average molecular weight; PDI: polydispersity index (*M*_w_/*M*_n_); ^3^ ++: Soluble; +−: partially soluble; −: insoluble; CHCl_3_: chloroform; THF: tetrahydrofuran.

**Table 3:** Comparison of optical properties of PI-3 and PS NFMs before and after thermal treatment.

Samples	*R*_457_ (%) ^1^	L* ^1^	a* ^1^	b* ^1^	*WI* ^1^
PI-1	90.4	91.02	−0.49	1.24	90.92
PI-2	91.3	91.49	−0.48	1.19	91.39
PI-3	90.2	91.38	−0.47	1.62	91.22
PI-4	89.5	90.94	−0.38	2.07	90.70
PI-ref ^2^	37.3	84.65	1.62	37.96	59.00
PI-50 °C	95.7	94.50	−0.43	3.51	93.46
PI-200 °C	89.0	93.29	−0.28	6.17	90.88
PS	88.4	93.52	−0.49	1.78	93.26
PS-50 °C	88.1	93.37	−0.46	1.79	93.23
PS-150 °C	18.1	ND ^3^	ND ^3^	ND ^3^	ND ^3^

^1^*R*_457_: Reflectance at a wavelength of 457 nm; *WI*: whiteness index; ^2^ PI-ref: poly(pyromellitic dianhydride-*co*-4,4′-oxydianiline) (PMDA-ODA) NFM derived from pyromellitic dianhydride (PMDA) and 4,4′-oxydianiline (ODA) [28]; ^3^ Not detected.

## Data Availability

Data are contained within the article.
